# Clustering and principal-components approach based on heritability for mapping multiple gene expressions

**DOI:** 10.1186/1753-6561-1-s1-s121

**Published:** 2007-12-18

**Authors:** Yuanjia Wang, Yixin Fang, Shuang Wang

**Affiliations:** 1 Department of Biostatistics, School of Public Health, Columbia University, 722 West 168th Street, New York, New York 10032, USA; 2 Department of Psychiatry-Behavioural Medicine, Columbia University, 1150 St. Nicholas Avenue, Suite 1-121, New York, New York 10032, USA

## Abstract

When the number of phenotypes in a genetic study is on the scale of thousands, such as in studies concerning thousands of gene expression levels, the single-trait analysis is computationally intensive, and heavy adjustment of multiple comparisons is required. Traditional multivariate genetic linkage analysis for quantitative traits focuses on mapping only a few phenotypes and is not feasible for a large number of traits. To cope with high-dimensional phenotype data, clustering analysis and principal-component analysis (PCA) are proposed to reduce the data dimensionality and to map shared genetic contributions for multiple traits. However, standard clustering analysis and PCA are applicable for independent observations. In most genetic studies, where family data are collected, these standard analyses can only be applied to founders and can lead to the loss of information. Here, we proposed a clustering method that can exploit family structure information and applied the method to 29 gene expression levels mapped to a reported hot spot on chromosome 14. We then used a PCA approach based on heritability applicable to small number of traits to combine phenotypes in the clusters. Lastly, we used a penalized PCA approach based on heritability applicable to arbitrary number of traits to combine 150 gene expression levels with the highest heritability. Genome-wide multipoint linkage analysis was carried out on the individual traits and on the combined traits. Two previously reported peaks on chromosomes 14 and 20 were identified. Linkage evidence was stronger for traits derived from methods that incorporate family structure information.

## Background

Gene expression levels, treated as complex quantitative traits, have been found to show familial aggregation [1]. Microarray technique allows measurement of thousands of gene expression levels simultaneously, providing an opportunity to map genetic determinants that regulate multiple expression levels. To locate such determinants, single-trait analysis can be performed on each individual trait and results can be compared [2]. However, when the number of gene expression phenotypes is on the scale of thousands, the single-trait analysis is computationally intensive, and heavy adjustment of multiple comparisons is required. Traditional multivariate genetic linkage analysis for quantitative traits focuses on mapping only a few phenotypes and is not feasible when the number of phenotypes is large [3]. To cope with high-dimensional phenotype data, clustering analysis and principal-component analysis were proposed to reduce dimensionality and to map shared genetic contributions for multiple traits [4]. However, the standard clustering analysis and principal-component analysis are applicable to independent observations. In most genetic studies, when family data are collected, these standard analyses are applied only to founders and can lead to loss of information [2].

Here, we proposed a clustering approach that takes into account of the family structure information. We then used a principal-components approach based on heritability proposed by Ott and Rabinowitz [5] to combine the phenotypes in each cluster. By maximizing the heritable component of the trait variation, this approach may increase power of linkage analysis on the combined trait because standard principal-components analysis may maximize non-genetic variance component [5,6]. The methods of Ott and Rabinowitz [5] are only applicable to a small number of phenotypes. We thus used a penalized principal components of heritability analysis proposed by Wang et al. [6] which can be applied to arbitrary number of traits to screen large number of expression levels simultaneously for hot spots. Genome-wide multipoint linkage analysis was applied to the first few combined traits.

## Methods

All analyses were performed on the GAW15 Problem 1 human gene expression data. Clustering analysis was applied to the 29 gene expression phenotypes found to have significant linkage results on chromosome 14 [2]. Principal-component analysis based on heritability [5] was performed to combine gene expression traits in each of the resulting cluster. A ridge-penalized principal-components approach based on heritability proposed by Wang et al. [6] was applied to the 150 gene expression levels with highest heritability. Multipoint linkage analysis was carried out on each of the 29 individual traits on chromosome 14 as well as on several combined traits.

### Clustering analysis

Here we proposed a clustering method that uses all subjects in the data set and incorporates family structure information by defining a distance measure that reflects similarity of traits among family members. This distance measure is a sum of weighted family-specific mean trait differences. The weights are calculated from within-family trait sum-of-squares. When the trait values for subjects within a family are more similar, leading to a smaller within-family sum-of-squares, the differences in their trait means is more important and thus is weighted larger. To be specific, let *i *index families and *j *index subjects. Let *n*_*i *_be the number of members in the *i*^th ^family. Then the distance between trait **x **and trait **y **is defined as

$$d(x,y)=\sum_{i}\frac{2(n_{i}-1){\left({\bar{x}}_{i.}-{\bar{y}}_{i.}\right)}^{2}}{\sum_{j}{\left(x_{ij}-{\bar{x}}_{i.}\right)}^{2}+\sum_{j}{\left(y_{ij}-{\bar{y}}_{i.}\right)}^{2}},$$

where ${\bar{x}}_{i.}=\sum_{j}x_{ij}/n_{i}$ and ${\bar{y}}_{i.}=\sum_{j}y_{ij}/n_{i}$. This distance measure resembles the ***F ***statistic in the ANOVA test. The proposed clustering using all subjects was compared to the standard hierarchical clustering using founders.

### Principal components of heritability

The principal-components approach based on heritability proposed by Ott and Rabinowitz [5] exploited family structure information by defining principal components of heritability (PCH) as scores with maximal heritability, subject to scores being orthogonal to each other. To be specific, a trait can be decomposed into a family-specific component and a subject-specific component. Instead of maximizing the total variation as in standard principal-components analysis, the PCH maximizes the relevant family-specific component variation relative to the subject-specific component variation. That is, the PCH is the solution to: $\underset{\alpha }{\max}\frac{\alpha^{T}B\alpha }{\alpha^{T}W\alpha }$, where *B *is the family-specific variation and *W *is the subject-specific variation. Note that this maximization criterion is equivalent to maximizing the heritability (the ratio of the family-specific variation to the total variation) of a score. Here we use between-family sum-of-squares to estimate *B*, and use within-family sum-of-squares to estimate *W*. The first three PCHs are computed in each of the clusters found in the previous section.

### Penalized principal-components of heritability

Without knowing which expression levels are regulated by a common gene, it may be desirable to apply the principal components of heritability approach on a large number of traits and evaluate which traits have significantly large loadings at linkage peaks. However, the method of Ott and Rabinowitz [5] is not applicable for high-dimensional traits for two reasons: first, it does not account for the problem of overfitting, which is a common problem to high-dimensional data; second, the sample within-family sum-of-squares (estimate of *W*) could be singular and cannot be inverted. Although generalized inverse can be used, the results will be highly unstable. In order to screen large number of traits, we used a penalized principal components of heritability [6] defined as

$$\underset{\alpha }{\max}\frac{\alpha^{T}B\alpha }{\alpha^{T}(W+\lambda I)\alpha }$$

to stabilize the PCH. Here, *λ *is the tuning parameter. When *λ *is zero, the penalized PCH is reduced to the PCH in Ott and Rabinowitz [5]; when *λ *approaches infinity, the penalized PCH approaches the score that maximizes the family-specific variation. In the latter case, the penalized PCH is close to the regular principal component applying to the founders. The *λ *is chosen by maximizing a cross-validated heritability [6]. We applied penalized PCH to 150 gene expression levels with the highest heritability.

### Linkage analysis

Prior to linkage analysis, genotype consistency was checked by PEDCHECK. SNPs with Mendelian genotyping errors were set to missing. Multipoint linkage analyses were performed by SIBPAL in S.A.G.E. The weighting method used for different sibling pairs was 'W4' [7]. The Rutgers genetic map provided by Sung et al. [8] was used. Linkage results from S.A.G.E. were summarized by *t *statistics and *p*-values.

## Results

### Clustering analysis

**Figure 1:**
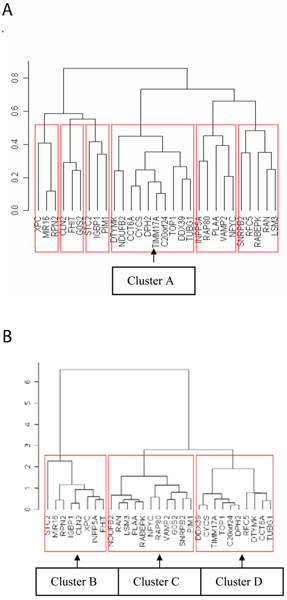
A, standard clustering; B, proposed clustering.

Standard hierarchical clustering computed from 56 founders is summarized in Figure 1a. The proposed family structure-based clustering computed from all subjects is summarized in Figure 1b. The first cluster tree was cut at 0.52, the threshold for correlation as suggested in Morley et al. [2], and the second tree was cut such that each cluster would have at least two members. Permutation can also be used to determine the cut-off value. The classical clustering produced six groups, while the proposed clustering produced three. Nine out of the ten members in the cluster A were in the cluster of 14 genes mapped to chromosome 14 hot spot reported by Morley et al. [2] compared to that, all the members in the cluster D were in the same cluster as reported in Morley et al. [2].

### Principal components of heritability

Principal components of heritability were computed for traits in Clusters A through D. Results were presented in Table 1. The first three components in Cluster A explained 72% of the total heritability. The corresponding proportion of heritability explained by the first three components in Cluster D was slightly higher (74%). The highest proportion explained was 77% in Cluster B.

**Table 1:** Principal components of heritability and linkage analysis

	Principal components analysis				Linkage analysis			
		
First 3 components in each cluster	Clustering method	Cluster	No. members	Cumulative % variation explained	Genome-wide peak *t*-value	Genome-wide peak *p*-value	SNP	Peak location
A.1	Classical	A	10	36%	3.7	1.25 × 10^-4^	rs1333050	chr 9 (43.9 cM)
A.2	Classical	A	10	58%	3.01	1.00 × 10^-3^	rs983795	chr 14 (77.3 cM)
A.3	Classical	A	10	72%	3.59	1.90 × 10^-4^	rs332364	chr 3 (93.3 cM)
B.1	Proposed	B	8	37%	4.31	1.06 × 10^-5^	rs941838	chr 14 (113 cM)
B.2	Proposed	B	8	62%	4.82	1.02 × 10^-6^	rs1955897	chr 14 (110.5 cM)
B.3	Proposed	B	8	77%	4.06	2.93 × 10^-5^	rs297675	chr 20 (11.9 cM)
C.1	Proposed	C	11	41%	4.46	5.31 × 10^-6^	rs1892302	chr 10 (27 cM)
C.2	Proposed	C	11	58%	4.99	4.62 × 10^-7^	rs2206185	chr 6 (111.2 cM)
C.3	Proposed	C	11	68%	5.36	7.35 × 10^-8^	rs1950475	chr 14 (109 cM)
D.1	Proposed	D	10	38%	4.61	2.78 × 10^-6^	rs1950475	chr 14 (109.6 cM)
D.2	Proposed	D	10	60%	3.32	4.95 × 10^-4^	rs739495	chr 19 (58.5 cM)
D.3	Proposed	D	10	74%	3.34	4.62 × 10^-4^	rs1955897	chr 14 (110.5 cM)

### Linkage analysis

**Figure 2:**
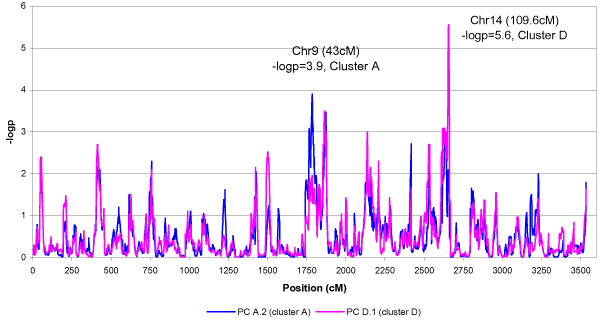
Linkage analysis of the principal components obtained from standard method (Cluster A) and proposed method (Cluster D).

Genome-wide linkage analysis was performed on each of the 29 expression levels mapped to chromosome 14. The maximum *t *value for the peaks reached 7.25, which corresponded to a *p*-value of 1.22 × 10^-12 ^and a genome-wide *p*-value of 3.53 × 10^-9 ^[9]. Genome-wide linkage scans for principal components of heritability are summarized in Table 1 and Figure 2. Note that each cluster had at least one component with a peak on chromosome 14. Among the components with a peak on chromosome 14, the linkage evidence was stronger for components derived from the proposed clustering method (Cluster B, C, and D) than the component derived from the standard method (Cluster A). For example, the peak *t *value for the component A.2 using the standard method was 3.01, while the peak *t *value for the component D.1, which shared several members with cluster A, was 4.61. Two components in Cluster B and one component in Cluster C mapped to the hot spot on chromosome 14 had *t *values 4.31, 4.82, and 5.35, respectively. The component C.2 showed a new peak on chromosome 6 with *t *value 4.99 (111.2 cM, Table 1).

### Penalized principal components of heritability

**Figure 3:**
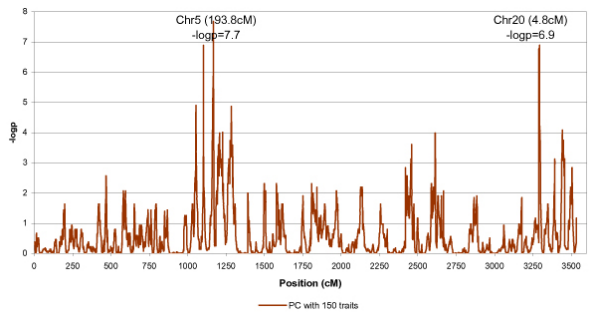
Linkage analysis of the penalized PCH approach applied to the 150 traits with highest heritability.

We applied the penalized PCH [6] to the 150 expression levels with the highest heritability. The cross-validation procedure suggested *λ *to be 7.88. Genome-wide linkage results of the first PCH were shown in Figure 3. There were two peaks on chromosomes 5 and 20, with *t *values of 5.60 (194 cM) and 5.25 (4.8 cM), respectively. The peak on chromosome 20 was also identified by Morley et al. [2].

## Discussion

We proposed a clustering method applicable to correlated family data. The distance measure used for clustering takes into account the trait similarity among family members. Unlike the standard hierarchical clustering, which only includes independent individuals, all the subjects in the data set contribute to the proposed method and can potentially recover some of the information lost in restricting analysis to founders. The clustering followed by PCH and multipoint linkage analysis identified the peak on chromosome 14 reported by Morley et al. [2]. The linkage evidence on chromosome 14 was stronger for the components computed from the proposed clustering (*p *= 7.35 × 10^-8^) than the ones computed from the standard clustering (*p *= 1.00 × 10^-3^). The penalized PCH approach applied to 150 traits with highest heritability identified a previously reported peak on chromosome 20 [2], suggesting it may be used to screen large number of traits for hot spot. However, note that the penalized PCH cannot be used to determine which traits to include for collinear traits. For example, for two perfectly correlated traits the cross-validation procedure cannot distinguish which trait is more important that the other without prior information.

Linkage analysis on the combined trait may give less significant results than on the individual trait after adjusting for multiple comparisons. This could be because the combined trait involves a linear combination of all traits, which is subjected to more noise. However, when the marginal effect of a gene on each trait is moderate but the combined effect is large, investigating single trait separately may not identify the gene, while a multivariate method could reveal the joint effect of such a gene. Another possible reason for less significant results of PCH might be that here we used within-family sum-of-squares to estimate subject-specific component variation and relatives' kinship relationship was not exploited. Such information can be added by incorporating kinship coefficients into a variance components model [10].

## Conclusion

The clustering analysis and the principal-components analysis based on family structure are useful to exploit available familial information in the gene expression family data. The linkage evidence is stronger using these methods than using the standard ones that ignores the family information. The penalized PCH approach can be applied to large number of traits. The cross-validation procedure can be used to select the optimal value of the penalty parameter.

## Competing interests

The author(s) declare that they have no competing interests.
